# Characterization of Clinical Isolates of *Enterococci* with Special Reference to Glycopeptide Susceptibility at a Tertiary Care Center of Eastern Nepal

**DOI:** 10.1155/2019/7936156

**Published:** 2019-07-01

**Authors:** Aasish Karna, Ratna Baral, Basudha Khanal

**Affiliations:** ^1^B.P. Koirala Institute of Health Sciences, Dharan, Nepal; ^2^Department of Microbiology and Infectious Diseases, B.P. Koirala Institute of Health Sciences, Dharan, Nepal

## Abstract

**Background:**

Enterococci, once considered as a harmless commensal of intestine, have now emerged as medically important pathogens and are associated with both community-acquired and nosocomial infections. They bear the potential to exhibit resistance against all commonly used antibiotics either by inherent or acquired mechanism, posing a therapeutic challenge.

**Objectives:**

This study aimed to characterize enterococci up to the species level and study their antibiogram with special regard to vancomycin.

**Methods:**

A descriptive cross-sectional study was conducted in the Department of Microbiology, B.P. Koirala Institute of Health Sciences, Dharan, Nepal, from February to May 2017. A total of 91 enterococcal isolates recovered from clinical specimens were investigated in this study. Their identification and speciation were done according to standard microbiological guidelines. Kirby–Bauer disc diffusion technique was used to study antimicrobial susceptibility pattern, whereas minimum inhibitory concentration of vancomycin was determined by the agar dilution method, with reference to Clinical and Laboratory Standards Institute guidelines.

**Results:**

Seven different species of enterococci were isolated, *E. faecalis* and *E. faecium* accounting about 45% each. The other species encountered were *E*. *avium*, *E*. *cecorum*, *E*. *dispar*, *E*. *durans*, and *E*.

**Conclusions:**

*Enterococcus faecalis* and *E. faecium* were the predominant species in causing enterococcal infections. The alarming rise in prevalence of vancomycin and multidrug resistance strains warrants immediate, adequate, and efficient surveillance program to prevent and control its spread.

## 1. Introduction

Enterococci are Gram-positive, facultatively anaerobic cocci that may occur in pair or short chains [1]. More recently, they are gaining greater attention because of their ability to withstand the effect of multiple antimicrobial agents, consequently limiting the therapeutic options and resulting in high mortality and morbidity. Tracking the distribution of enterococci and the knowledge of their antibiogram is of utmost importance in order to prevent and control its spread [2]. They bear the potential to cause a wide range of infections, for instance, urinary tract infections, surgical site infection, bacteremia, intra-abdominal and intra-pelvic abscess, and occasionally, meningitis and pneumonia [3, 4]. Enterococci exhibit low-level resistance to all aminoglycosides, indicating poor efficacy in clinical practice. The predisposing factors associated with rise in enterococcal infections are empirical use of antimicrobial agents, prolonged hospitalization, invasive therapy, and wide use of immunosuppressant [2, 5, 6]. Their capability of transferring determinants of antibiotic-resistant genes between the species, as well as other bacteria (*Staphylococcus aureus*), is a global cause of concern [7]. Enter*ococci* can be disseminated easily through different routes of transmissions such as contaminated water, infected animal meat, inanimate objects, and hands of healthcare workers [7–9], that has resulted in the widespread of enterococcal infections, which further is exacerbated by their ability to tolerate a wide range of temperature and pH, high salt concentration, and some alcohol solutions [7].

Identification and speciation of enterococcal isolates possess substantial impact on therapeutic choice since antimicrobial susceptibility pattern varies between the species. Even though vancomycin-resistant enterococci (VRE) were first reported in 1986, from the UK and France, in recent years, they have been found to be disseminated all around the world [10]. VRE have risen markedly by 20-folds in the last two decades, with the recovery rate varying between 0% and 35%, internationally [11]. The incidence of vancomycin-resistant*E. faecium* in Asia is yet low; nevertheless, outbreaks have been reported [7]. In Nepal, limited studies have been undertaken on prevalence of enterococci in the clinical environment and paucity of data is available on VRE. In the previous study from our own center between 2002 and 2003, the prevalence of VRE among *E. faecalis* and *E. faecium* were documented to be 13% and 17%, respectively [12]. Similarly, a single strain of vancomycin-resistant*E. faecium* was reported from a case study of a patient with peritonitis [13]. Likewise, 4 isolates out of 41 were found to be vancomycin intermediate by the agar dilution method, in another medical college [14].

This study was undertaken with the objective to characterize enterococci up to the species level and study their antibiogram with special regard to vancomycin.

## 2. Materials and Methods

### 2.1. Study Design and Area

A descriptive cross-sectional study was conducted in the Department of Microbiology, B.P. Koirala Institute of Health Sciences, Dharan, Nepal, from February to May 2017. Consecutive sampling technique was used. A total of 91 enterococcal isolates recovered from various clinical samples submitted to Microbiology Laboratory for routine culture and susceptibility testing during the study period were analyzed in this study.

### 2.2. Isolation and Identification of Enterococcal Isolates

All the clinical specimens such as blood, pus, aspirates, wound swab, high vaginal swab, and peritoneal fluid were inoculated on to Blood and MacConkey agar, whereas urine sample was plated on to cysteine-lactose-electrolyte-deficient medium. The culture plates were incubated at 35°C for 24–48 hours. Brain heart infusion broth was used for all blood samples. After 24 and 48 hours of incubation, subculture of the blood sample was performed as for the other specimens. Growth was noted on the following day. Enterococci were identified with reference to colony morphology, Gram staining, catalase test, bile-esculin test, and salt tolerance test (6.5% NaCl). Further speciation was done on the basis of pigment production, motility test, and biochemical reactions as per standard microbiological guidelines [15, 16].

### 2.3. Antimicrobial Susceptibility Testing

Antimicrobial susceptibility testing of enterococcal isolates was performed by the disc diffusion technique (DDT) according to Clinical and Laboratory Standards Institute (CLSI) guidelines against penicillin (10 units), ampicillin (10 *μ*g), vancomycin (30 *μ*g), teicoplanin (30 *μ*g), ciprofloxacin (5 *μ*g), chloramphenicol (30 *μ*g), linezolid (30 *μ*g), and high-level gentamicin (120 *μ*g) and in addition, nitrofurantoin (300 *μ*g) for urinary isolates. With the help of a straight inoculating wire, 3–5 well-isolated colonies of the same morphology were picked and transferred into a tube containing 5 ml of Mueller Hinton broth. The broth culture was incubated at 35°C until the turbidity of 0.5 McFarland standard was achieved. The comparison was done visually in adequate light against a card with a white background and contrasting black lines. After the turbidity was gained, a sterile cotton swab was dipped into inoculum suspension within 15 minutes. Any excess inoculum from the swab was removed by rotating and firmly pressing it on the inside wall of the tube above the fluid level. The swabs were lawn cultured over the entire surface of dried sterile Mueller Hinton agar plate. Thereafter, antimicrobial discs supplied by HiMedia Laboratories, India, were dispensed onto the surface of inoculated agar plate. The plates were inverted and incubated within 15 minutes at 35°C for 16–18 hours. Eventually, the result was read and interpreted [17].

### 2.4. Determination of Minimum Inhibitory Concentration of Vancomycin

Minimum inhibitory concentration (MIC) of vancomycin was determined according to the agar dilution method. Brain heart infusion agar media and vancomycin powder of potency 950 *μ*g/mg obtained from HiMedia Laboratories, India, were used in the process. Procedures were undertaken as recommended by standard microbiological guidelines, and results were reported with reference to CLSI guidelines. The lowest concentration of antibiotic, inhibiting visible growth after recommended incubation, was regarded as MIC [17, 18].

### 2.5. Discrepancies between the Methods for Detection of Vancomycin Resistance

Errors have been found to be associated with DDT in assessing vancomycin resistance among enterococcal isolates and are categorized as “very major error,” “major error,” and “minor error” [19]. If a strain susceptible by the test method exhibit resistance to the standard agar/broth reference method, then the error is called very major error. In contrast, major error is defined as if a strain resistant by the test method appears to be susceptible by the agar/broth reference method. Likewise, an intermediately susceptible strain by either of the methods showing either resistance or susceptibility pattern by the other method is regarded as minor error [19, 20].

### 2.6. Data Management and Statistical Analysis

Collected data were entered in Microsoft Excel 2016 and analyzed by Statistical Package for Social Sciences (SPSS, version 11.5). Descriptive statistics was calculated and summarized (frequency, rate, ratio, proportion, percentage, and cross tabulation). Cohen's kappa coefficient (*K*) statistics was used to measure the agreement between the disc diffusion technique and agar dilution method in determining vancomycin resistance. *K* value > 0.8 was considered as “very good” agreement. According to Cohen, *K* value > 0.8 and < 0.2 is, respectively, indicated to have “very good” and “poor” level of concordance. Likewise, *K* statistics in between 0.61–0.80, 0.41–0.60, and 0.21–0.40 represents good, moderate, and fair agreement, respectively. The chi-squared test was used to compare our findings with a *p* value ≤ 0.05 indicative of statistical significance.

## 3. Results

A total of 91 strains of enterococci recovered from different clinical specimens were analyzed in this study. Among these isolates, 61.5% (56) were obtained from hospitalized patients, 8.8% (8) from emergency department, and 29.7% (27) were from outpatient department. The ratio of male to female in the study participants was 1 : 1. A majority of isolates were obtained from 0–10 age group (20.9%), followed by an age bar of 20–30 (19.8%). Details of type of specimens recovered from various age groups are depicted in Table 1.

Based on the standard guideline of species identification [15, 16], seven different species of enterococci were identified. *E. faecalis* and *E. faecium* together accounted for more than 90% of total isolates. Highest frequency of isolates was recovered from urine (61.5%), followed by pus (19.8%) and blood (5.5%). Nature of specimen from which enterococcal isolates were recovered is illustrated in Table 2.

Resistance of *E. faecium* strains was higher than that of *E. faecalis* against all antimicrobial agents tested except chloramphenicol and vancomycin. None of the isolates of *E. cecorum*, *E. dispar*, and *E. durans* were resistant to any of the antibiotics tested. Highest frequency of susceptibility was observed for linezolid (97.8%), followed by teicoplanin (95.6%) and high-level gentamicin (81.3%). Forty-six of 56 (82.1%) urinary isolates were susceptible to nitrofurantoin. Antimicrobial resistance profile of enterococcal isolates is shown in Table 3.

A total of seventy-two isolates were found to be susceptible to vancomycin by DDT; however, 4 strains among susceptible isolates were grown on agar plate containing vancomycin of concentration 8 mg/L, and was categorized as minor error. Even though, kappa agreement between DDT and agar dilution method was strong (*K* = 0.88, *p* ≤ 0.01), DDT failed to recognize reduced susceptibility to vancomycin in 4 enterococcal isolates.

Out of 91 enterococcal isolates, 23 (25.3%) were found to be VRE by the agar dilution method, of which MIC of vancomycin against 13 *E. faecalis*, 5 *E. faecium*, and 1 *E. avium* was 8–16 mg/L, and were considered as vancomycin intermediately resistant, whereas remaining 4 VRE were grown on agar plate containing vancomycin of concentration 256 mg/L and consequently were regarded as high-levelvancomycin-resistant (MIC ≥ 32 mg/L). All those 4 high-level resistant VRE were identified as *E. faecium*. Overall, prevalence of vancomycin-resistant*E. faecalis* was higher than of vancomycin-resistant*E*. *faecium*. Nevertheless, only *E. faecium* species exhibited the highest level of resistance against vancomycin. MIC observed in different enterococcal isolates is summarized in Table 4.

Among 91 strains of enterococci, 29.7% (27) did not exhibit resistance against any of the antibiotics tested. However, multidrug resistance (MDR) was noted in 31.9% (29) of isolates. Prevalence of MDR was highest in *E. faecium* 62% (18/29). *E. avium* also showed resistance to three antimicrobial classes. The prevalence of MDR and their resistance pattern are depicted in Table 5.

## 4. Discussion

The rapid emergence of resistant *Enterococcus* species are associated with major health threats globally. The current practice of empirical therapy in different parts of eastern Nepal and across the border is seemingly ruining many antibiotic treatment regimens. Patients visiting from these areas to our referral center tend to use antibiotics irrationally prior to their visit for treatment. This might be because of unavailability of proper microbiology diagnostic laboratory at their primary care center. The other cause probably could be due to lack of regulation to purchase antibiotics without a medical prescription or lack of awareness among them about the outcome of irrational use of antibiotics. This study investigated the occurrence of enterococcal species from various clinical specimens and antimicrobial susceptibility pattern exhibited by them.

We did not experience significant dominance of *E. faecalis* over *E. faecium* in the current study. Similar finding was also reported from Brazil [4]. In majority of studies, *E. faecalis* have been reported to be the predominant enterococcal species, followed by *E. faecium* [3, 5, 21]. The increased proportion of *E. faecium* in our study may be because of their ability of attaining resistance against multiple antibiotics. Unlike above studies, we did not come across *E. gallinarum*, *E. caseliflavus*, *E. solitarius*, and *E. hirae.*

In the current study, 91 enterococcal isolates were recovered from 5652 clinical specimens within the study period of four months. However, in a similar study conducted in our own center between March 2002 and February 2003, only 50 enterococcal isolates were recovered from 8627 clinical specimens [12]. This indicates that enterococci have significantly (*p* ≤ 0.05) risen over the years as one of the leading causes of human infection. Unlike previous study, four new species of *Enterococcus* were recovered, namely, *E. cecorum*, *E. dispar*, *E. durans*, and *E*. *raffinosus*. Likewise, it was noted that *E. faecium* are seemingly changing the pattern of enterococcal infection, with increase in their isolation rate. The recovery ratio of enterococci from hospitalized patients and outpatient department was almost similar in both studies. Nevertheless, a majority of isolates in this study were obtained from urine (56/91), followed by pus (18/91) and blood (5/91), which is in contrary to blood (15/50) and pus (15/50) being the major source of enterococcal recovery in the previous report. The overall prevalence of VRE in our study was double (25.3%) than that of the study undertaken about fifteen years back (11.7%). The occurrence proportion of MDR was highest in *E. faecium* in both studies. Over the years, resistance against vancomycin and ciprofloxacin was found to be increased in contrast to chloramphenicol and ampicillin. The increased frequency of resistance against vancomycin and ciprofloxacin in our study might be because of irrational use of these antibiotics or probably due to variation in sample size. Likewise, in a case report from our institution published in 2014 [13], a high level of vancomycin resistance was observed in *E. faecium*, which lines up with our finding.

In the present study, enterococci tested against commonly used antibiotics by the Kirby–Bauer disc diffusion method showed highest susceptibility for linezolid (97.8%), followed by teicoplanin (95.6%), high-level gentamicin (81.3%), and vancomycin (79.1%), which was similar to picture in other studies except, higher incidence of high-level gentamicin resistance (HLGR) than vancomycin resistance was noted in the previous studies [2, 22]. Low prevalence of resistance against vancomycin, teicoplanin, and linezolid was observed in a study from China [6]. A total of 4 isolates in our study exhibited teicoplanin resistance. All those teicoplanin-resistant strains were *E. faecium* and were also resistant to vancomycin. Similar finding was also reported from Mumbai, India [23], whereas higher prevalence of teicoplanin-resistant enterococci were reported from another state of India [1].

In our study, prevalence of ampicillin- and penicillin-resistant pattern was about 40%, each. The finding is more likely agreed with report from Bangalore, India [24]. However, lower and higher prevalence of beta-lactam resistance was reported from West Bengal [25] and Bihar [26] states, respectively. The resistance rate to beta-lactam was found to be more common in *E. faecium* than *E. faecalis*, which agreed with other studies [6, 27]. The reason could be lower affinities between these antibiotics and penicillin-binding protein (PBP) of *E. faecium* and/or presence of plasmid-encoded* β*-lactamase in some strains [28]. Other study has reported that resistance exhibited by enterococcal isolates against *β*-lactam was up to 95% [2].

In the current study, HLGR was observed in 18.7% of enterococcal isolates by DDT that is comparable with result documented from Kuwait, 14% [29]. However, the incidence of HLGR enterococci was found to be higher in a study by Bhatt et al., 65% [2], and Dadfarma et al., 43.7% [5]. This might be due to implication of different techniques in accessing high-level resistance against gentamicin from our study; in the previous studies, HLGR was reported on the basis of minimum inhibitory concentration, by the *E*-test and microdilution method, respectively. Among 17 HLGR in our study, 82% (12 *E. faecium* and 2 *E. faecalis*) were found to be associated with *β*-lactam resistance. In the current study, concomitant resistance percentage of HLGR to *β*-lactam was higher than of reports from northern Tehran, 61.3% [5], which might be due to greater proportion of *E. faecium* in our study than previous one. Such accompanying strains have potential of destroying synergistic effect of aminoglycosides and *β*-lactam combination, consequently hindering the therapy against enterococcal infections. Thus, early steps must be taken before it results into pandemic.

The prevalence of chloramphenicol resistance in our study was 23.1%. This finding is in close proximity with the literature from Saudi Arabia, 22.7% [27]. The present study showed resistance percentage exhibited by *E. faecalis* to chloramphenicol than *E. faecium* was higher, which lines up with other studies [3, 6]. Chloramphenicol has received attention as a therapeutic option for treating infections caused by VRE. There was no significant difference in the chloramphenicol resistance rate with the previous report published from our own institution in 2007 [12]. Significant association (*p* value ≤ 0.05) between VRE and chloramphenicol-sensitive enterococci was noted in current study. This finding indicates that chloramphenicol can be used as drug of utmost importance against VRE, which agreed with other studies [30, 31]. Therefore, it should not be used indiscriminately.

On the other hand, the incidence of ciprofloxacin-resistant enterococci in our study was 61.5%. This result is similar with the finding documented from Saudi Arabia, 61.9% [27]. Out of 56 urinary isolates, 76.7% isolates were resistant to ciprofloxacin. However, a higher and lower prevalence of ciprofloxacin resistant strains among urinary isolates was observed in other studies, respectively [1, 22]. The high resistance to ciprofloxacin probably could be because of its irrational usage as broad-spectrum antibiotic against a wide range of diseases. Enterococci are considered as the second most leading cause of hospital-acquired urinary tract infections [24]. Resistance against nitrofurantoin was noted in 17.9% of urinary isolates. This finding is lower than a report from Iran, 25.4% [32]. The lower prevalence of nitrofurantoin resistance in our study indicates that it can be used as therapeutic option in urinary tract infections caused by enterococcal species.

The overall prevalence of VRE in our study was found to be 25.3%. Similar to our study, incidence of VRE in Iran was also reported, 21.4% based on MIC of vancomycin [32]. Nevertheless, this result is lower than other reports, 39.6% [33], and is higher than other studies [23, 34]. Such difference in prevalence may be due to variation in geographical location, sample size, duration of hospital stays, and use of invasive devices. Among the 23 VRE strains, 4 *E. faecium* showed high-level resistant against vancomycin (MIC = 256 mg/L) and were obtained from urine sample of patients at extreme age (1-pediatric, 3-geriatrics). *E. faecium* were significantly (*p* value ≤ 0.05) found to exhibit high-level resistance (MIC ≥ 32 mg/L) against vancomycin in the present study, which is in contrast to other reports [32], in which *E. faecium* were not reported to have significant association with high-level MIC of vancomycin among resistant strains, indicating the potentiality of both *E. faecalis* and *E. faecium* to cause high-level vancomycin resistance. The difference might be because of lower sample size in our study than their study. Even though significant difference in enterococcal infection with gender was not seen, male preponderance (73.9%, 17/23) was observed with vancomycin resistance, which might be due to greater proportion of male patients being admitted than female. Highest frequency of VRE strains (21.7%) was recovered from each surgery and pediatric ward. This picture is in concurrence with the result from Bihar, India [26], in which the VRE recovery rate was more in surgery department and majority of males were found to be colonized with VRE. Overall, we did not experience significant association between enterococcal species and vancomycin-resistant strains; in contrast, many studies reported *E. faecium* to be the predominant species to exhibit resistance against vancomycin.

Of the total enterococcal isolates, 31.9% showed MDR, which was defined by resistance to one or more agents in at least three antimicrobial categories [35]. The proportion of MDR was found to be more prevalent among *E. faecium*(18/41), as compared to *E. faecalis*(10/42) and HLGR (14/17) than non-HLGR(15/74) and in vancomycin resistance (12/23) in contrast to vancomycin-sensitive strains (17/68). This result is more likely comparable with the other studies [2, 5, 36], in which higher prevalence rate of MDR was observed among *E. faecium*, HLGR, and VRE, respectively. However, the overall incidence of MDR in our setting was lower than that in other studies [2, 5, 36].

## 5. Conclusion

Our study outlines the soaring prevalence of *E. faecium* in the clinical specimens, which is alarming since it is more likely to surpass *E. faecalis* in causing enterococcal infections. The overall prevalence of VRE and MDR in our study was found to be 25.3% and 31.9%, respectively. This picture demonstrates high incidence of VRE and MDR strains in our setting. Therefore, regular surveillance and efficient infection control program must be implemented in order to prevent its dissemination. Moreover, antibiotic stewardship must be strictly followed to minimize antimicrobial resistance. Due to fallacy of DDT in detecting vancomycin resistance among enterococcal isolates and because of resource constraints, the *E* test is not available therefore, the agar dilution method is recommended for laboratory screening and monitoring of VRE.

## Figures and Tables

**Table 1:** Nature of specimens recovered from different age groups.

Age group in years	Types of specimens											Total
	Ascitic fluid	Bile	Blood	Endotracheal tube	High vaginal swab	Peritoneal fluid	Pus	Semen	Tissue	Urine	Wound swab	
0–10	0	0	4	0	0	0	2	0	1	12	0	19
10–20	0	0	0	0	0	1	2	0	0	5	2	10
20–30	1	0	0	1	0	0	3	0	1	11	1	18
30–40	0	0	1	0	1	0	6	1	1	4	0	14
40–50	0	1	0	0	0	0	1	0	0	8	0	10
50–60	0	0	0	0	0	0	1	0	0	7	0	8
>60	0	0	0	0	0	0	3	0	0	9	0	12
Total	1	1	5	1	1	1	18	1	3	56	3	91

**Table 2:** Distribution of *Enterococcus* species in various clinical specimens.

Specimens	*E. avium*	*E. cecorum*	*E. dispar*	*E. durans*	*E. faecalis*	*E. faecium*	*E. raffinosus*
Ascitic fluid (*n* = 1)	0	0	0	0	0	1	0
Bile (*n* = 1)	0	0	0	0	0	1	0
Blood (*n* = 5)	0	0	0	0	1	4	0
Endotracheal tube (*n* = 1)	0	0	0	0	1	0	0
High vaginal swab (*n* = 1)	0	1	0	0	0	0	0
Peritoneal fluid (*n* = 1)	0	0	0	0	0	0	1
Pus (*n* = 18)	1	0	1	1	6	7	2
Semen (*n* = 1)	0	0	0	0	1	0	0
Tissue (*n* = 3)	0	0	0	0	2	1	0
Urine (*n* = 56)	0	0	0	0	29	27	0
Wound swab (*n* = 3)	0	0	0	1	2	0	0
Total (*n* = 91)	1	1	1	2	42	41	3

**Table 3:** Antimicrobial resistance patterns among enterococcal species (N (%)).

Antibiotics	*E. avium* (*n* = 1)	*E. cecorum* (*n* = 1)	*E. dispar* (*n* = 1)	*E. durans* (*n* = 2)	*E. faecalis* (*n* = 42)	*E. faecium* (*n* = 41)	*E. raffinosus* (*n* = 3)	Total (*n* = 91)
Penicillin	1 (100)	0	0	0	7 (16.7)	28 (68.3)	1 (33.3)	37 (40.7)
Ampicillin	1 (100)	0	0	0	7 (16.7)	27 (65.9)	1 (33.3)	36 (39.5)
Vancomycin	1 (100)	0	0	0	11 (26.2)	7 (17.1)	0	19 (20.9)
Teicoplanin	0	0	0	0	0	4 (9.8)	0	4 (4.4)
Ciprofloxacin	1 (100)	0	0	0	27 (64.3)	28 (68.3)	0	56 (61.5)
Chloramphenicol	0	0	0	0	14 (33.3)	7 (17.1)	0	21 (23.1)
High-level gentamicin	0	0	0	0	5 (11.9)	12 (29.3)	0	17 (18.7)
Linezolid	0	0	0	0	0	2 (4.9)	0	2 (2.2)
Nitrofurantoin	0	0	0	0	3 (10.3)	7 (25.9)	0	10 (17.9)^1^

^1^Only urinary isolates were tested against nitrofurantoin, and 17.9% (10/56) strains were resistant to it.

**Table 4:** MIC value of vancomycin (*μ*g/ml) in different clinical isolates.

Species of *Enterococcus*	MIC values (mg/L)								
	≤1	2	4	8	16	32	64	126	256
*E. avium* (*n* = 1)	0	0	0	1	0	0	0	0	0
*E. cecorum* (*n* = 1)	0	1	0	0	0	0	0	0	0
*E. dispar* (*n* = 1)	0	1	0	0	0	0	0	0	0
*E. durans* (*n* = 2)	0	1	1	0	0	0	0	0	0
*E. faecalis* (*n* = 42)	0	6	23	13	0	0	0	0	0
*E. faecium* (*n* = 41)	0	5	27	3	2	0	0	0	4
*E. raffinosus n* = 3)	0	1	2	0	0	0	0	0	0
Total (*n* = 91)	0	15	53	17	2	0	0	0	4

**Table 5:** Multidrug resistance profile of *Enterococcus*.

Enterococcal species	No. of isolates exhibiting multidrug resistant pattern		
	R3	R4	R5
*E. faecium*	8	5	5
*E. faecalis*	6	2	2
*E. avium*	1	0	0
Total MDR (*N*, (%))	15 (16.5)	7 (7.7)	7 (7.7)

R3 = resistant to 3 antimicrobial classes, R4 = resistant to 4 antimicrobial classes, R5 = resistant to 5 antimicrobial classes, and MDR = multidrug resistance.

## Data Availability

The data used to support the findings of this study are available from the corresponding author upon request.

## Abbreviations

VRE: Vancomycin-resistant enterococci
DDT: Disc diffusion technique
CLSI: Clinical and Laboratory Standards Institute
MIC: Minimum inhibitory concentration
K: Cohen's kappa coefficient value
MDR: Multidrug resistance
HLGR: High-level gentamicin resistance.
